# IRF-1 contributes to the pathological phenotype of VSMCs during atherogenesis by increasing CCL19 transcription

**DOI:** 10.18632/aging.202204

**Published:** 2020-11-16

**Authors:** Yongbin Shen, Zhanfeng Sun, Shuran Mao, Yingnan Zhang, Weiliang Jiang, Haitao Wang

**Affiliations:** 1 Department of Vascular Surgery, The 2nd Affiliated Hospital of Harbin Medical University, Harbin 150086, China; 2 Department of Plastic Surgery, The 2nd Affiliated Hospital of Harbin Medical University, Harbin 150086, China

**Keywords:** IRF-1, VSMCS, atherosclerosis, CCL19

## Abstract

Atherosclerosis (AS) is a chronic inflammatory disease that mainly involves the large and middle arteries, but the specific mechanism is not precise. Chemokine ligand 19 (CCL19) has been reported highly expressed in peripheral blood of patients with atherosclerosis, but its role lacks explicit data. By ELISA assay and immunohistochemical (IHC) analysis, we found that the CCL19 was significantly up-regulated in AS. Therefore, we tried to clarify whether CCL19 expression was related to the progression of AS. QRT-PCR and western blot demonstrated that overexpression of CCL19 promoted the secretion of inflammatory factors and the deposition of the extracellular matrix, and facilitated the proliferation and migration of VSMCS. Besides, knockdown of CCL19 reduced the inflammation, collagen secretion, proliferation and migration of VSMCS induced by PGDF-BB. The results of database analysis, chromatin immunoprecipitation (ChIP) and luciferase assay showed that interferon regulatory factor 1 (IRF-1) activated the expression of CCL19 at the transcriptional level. Importantly, silencing IRF-1 inhibited atherosclerosis in high-fat-fed mice, inhibited the proliferation and migration of VSMCS, and down-regulated the expression of CCL19. Summing up, the results demonstrated that IRF-1 contributed to the pathological phenotype of VSMCs during atherogenesis by increasing CCL19 transcription.

## INTRODUCTION

Atherosclerosis (AS) is a chronic vascular inflammatory disease affected by multiple factors [1]. Lipids deposited on the wall of inflammatory damaged vessels, and with the infiltration of inflammatory cells, atherosclerotic plaques are gradually formed [2]. Radio-tracers that target atherosclerotic plaque inflammation are helpful for imaging detection for the evaluation of patients [3]. Plaque rupture and content release lead to the formation of arterial thrombus, resulting in malignant clinical events such as myocardial infarction, coronary heart disease and ischemic stroke [4].

Vascular smooth muscle cells (VSMCs) are the main cell and highly differentiated cells that make up the vascular wall tissue. The proliferation, migration and senescence of VSMCS affect the formation and development of AS. In the process of AS formation, medial VSMCS proliferate massively under the stimulation of inflammatory factors and chemokines, and migrate from media to intima, synthesize collagen and other extracellular stroma, and transform lipids into foam cells, resulting in thickening of vascular wall, increase of foam cells and formation of lipid plaques on vascular wall [5, 6].

Chemokine ligand 19 (CCL19) is a vital chemokine, which performs a series of biological functions including chemotaxis by binding to chemokine receptor 7 (CCR7) [7]. Akhavanpoor M et al. [8] have reported that the expression of CCL19 and CCL21 in atherosclerotic lesions increased and may represent a potent immunoregulatory treatment approach. In intimal medullary cells, reverse transendothelial migration dependent on CCR7 and CCL19 protect normal arterial intima during atherosclerosis [9]. However, the effect of CCL19 on VSMCS has not been reported.

Interferon regulatory factor 1 (IRF-1) was the first identified and the most deeply explored transcriptional regulator of the IRF family. It was initially regarded as a transcriptional regulator of IFN-α/β and played an important role in congenital and acquired immune responses [10], which exerts anti-tumor effect by activating apoptosis signal pathway [11]. Du M et al. have demonstrated that silencing IRF-1 alleviate atherosclerosis in mice by regulating lipid metabolism and foam cell formation, and suggest that IRF-1 activation is a risk factor for the occurrence and development of atherosclerosis [12].

The purpose of this study is to clarify the role of CCL19 in extracellular matrix (ECM) accumulation, proliferation, migration and inflammation of vascular smooth muscle, and to further explore the mechanism of IRF-1 and the transcriptional regulation on CCL19 during AS formation.

## RESULTS

### CCL19 is upregulation in AS

**Figure 1:**
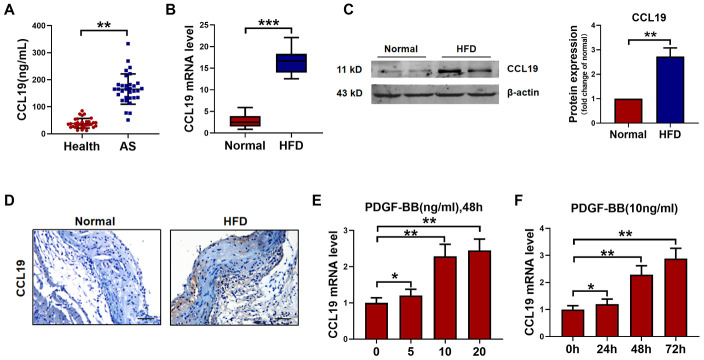
**CCL19 expression in AS.** (**A**) Detection of CCL19 expression in peripheral blood of patients by ELISA. n=32. (**B**) qRT-PCR analysis of CCL19 mRNA levels normalized to GAPDH in AS mice aorta and normal aorta tissues. n=10. (**C**) CCL19 protein levels in the AS or normal mice aorta tissues was performed by western blot. n=4. (**D**) Representative images of IHC of CCL19 in AS model mice aorta tissues and normal aorta tissues. Bar=50 μm. The effect of different concentrations (**E**) or different duration (**F**) of PDGF-BB on the expression of CCL19 was detected by qRT-PCR. n=5. *P<0.05; **P<0.01; ***P<0.001.

The serum level of CCL19 in peripheral blood of AS patients was higher than that of healthy controls by ELISA (Figure 1A). The mRNA level of CCL19 in the descending aorta of ApoE-/- mice was detected by qRT-PCR. It was found that the CCL19 mRNA level of AS mice was higher than normal feeding group (Figure 1B). The protein level of CCL19 was higher in AS mice artery detected by western blot and Immunohistochemical staining (Figure 1C, 1D). The abnormal proliferation of VSMCs is one of the important reasons for the occurrence and development of AS. Platelet-derived growth factor (PDGF) plays an essential role in cardiovascular disease, which can lead to chronic inflammatory remodeling and atherosclerosis. By detecting different concentrations of recombinant PDGF factor at 48h, we found that 10ng/mL PDGF-BB could significantly induce the high expression of CCL19 in VSMCS cells (Figure 1E), At the same time 48h had a more apparent stimulating effect on CCL19 (Figure 1F).

### CCL19 functionally promotes VSMCS inflammation, collagen deposition, proliferation and migration

**Figure 2:**
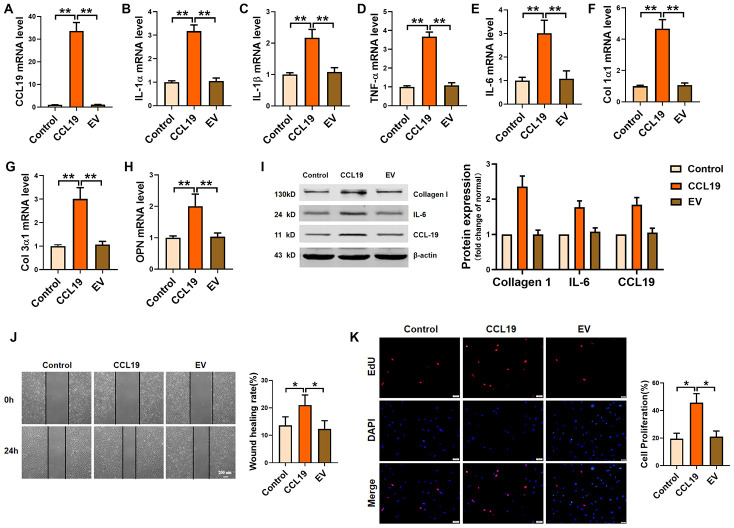
**Effects of CCL19 on VSMCS inflammation, ECM deposition, proliferation and migration.** (**A**) Detection of overexpression efficiency of CCL19 plasmid was measured by qRT-PCR. The inflammatory cytokine IL1-α (**B**), IL-1β (**C**), TNF-α (**D**), IL-6 (**E**) and the deposition of ECM such as collagen1 (**F**), collagen3 (**G**) and OPN (**H**) were detected by qRT-PCR.n=5 (**I**) Western blot showed the expression of proteins collagen1 and IL-6. n=4. Migration (**J**) and proliferation (**K**) of VSMCS after overexpressing CCL19. n=6. EV: Empty vector. *P<0.05; **P<0.01.

In order to determine the effect of CCL19 on VSMCS, we constructed a CCL19 overexpression plasmid and verified the overexpression efficiency of the plasmid (Figure 2A). QRT-PCR results certified that forced expression of CCL19 promoted the secretion of inflammatory factors in VSMCS cells, including IL-1 α, IL-1 β, TNF- α and IL-6 (Figure 2B–2E). The extracellular matrix levels such as Collagen1 and Collagen3 and osteopontin (OPN) were also increased (Figure 2F–2H). Western blot results showed that overexpression of CCL19 could significantly increase the protein expression of Collagen1 and IL-6 (Figure 2I). Excessive proliferation and migration of VSMCS is also a major pathological phenomenon in the process of atherosclerosis. We found that CCL19 can promote the migration (Figure 2J) and proliferation (Figure 2K) of VSMCS.

### Silencing CCL19 protects against the pathological phenotype of VSMCS induced by PDGF-BB

**Figure 3:**
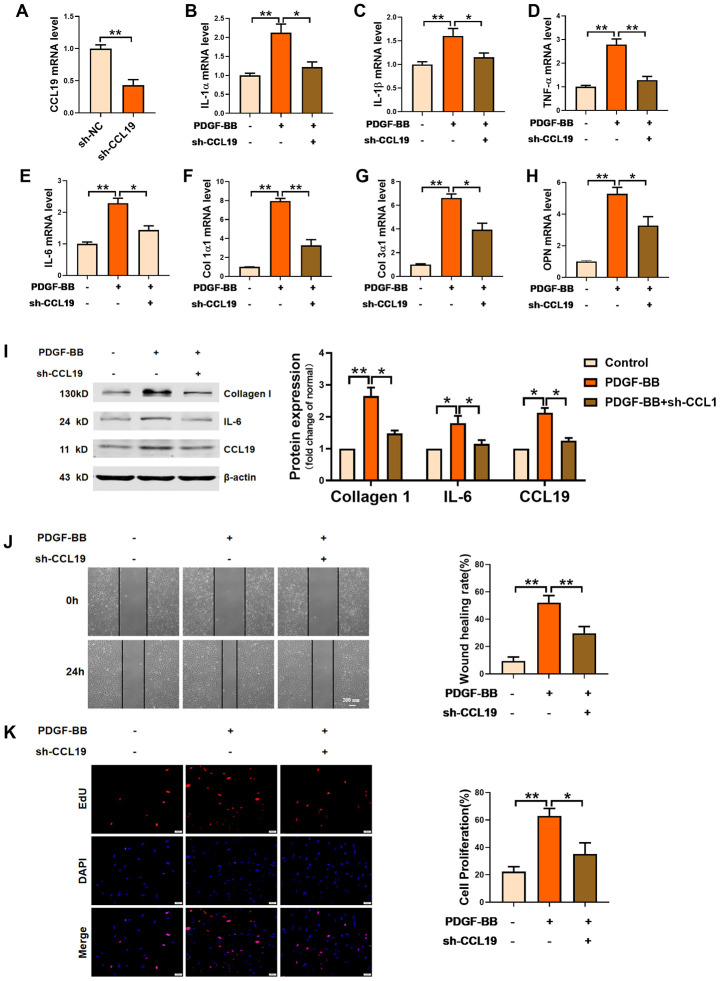
**Effects of silencing CCL19 on VSMCS. Detection of silencing efficiency of sh-CCL19 plasmid was detected by qRT-PCR.** (**A**) Knockdown of CCL19 inhibited mRNA level of IL1-α (**B**), IL-1β (**C**), TNF-α (**D**), IL-6 (**E**) and collagen1 (**F**), collagen3 (**G**) and OPN (**H**) induced by PDGF-BB.n=5 (**I**) Western blot show collagen1 and IL-6 protein expression. n = 4. Migration (**J**) and proliferation (**K**) of VSMCS after silencing CCL19. n = 6. *P<0.05; **P<0.01.

Furthermore, we constructed a short hairpin RNA for silencing CCL19 and verified the silencing efficiency of the plasmid (Figure 3A). RT-PCR results showed that sh-CCL19 inhibited the secretion of IL-1 α, IL-1 β, TNF- α and IL-6 induced by PDGF-BB in VSMCS (Figure 3B–3E). The levels Collagen1 and Collagen3 and osteopontin OPN was also decreased (Figure 3F–3H). Western blot results showed that sh-CCL19 significantly reduced the protein expression of Collagen1 and IL-6 (Figure 3I). We found that sh-CCL19 could weaken the ability of migration and proliferation of VSMCS (Figure 3J, 3K). These results suggested that the high expression of CCL19 in the atherosclerosis may be a blood biomarker for predicting AS, and the high expression of CCL19 could promote the progression of AS.

### CCL19 is the direct target of IRF-1 in VSMCS abnormal lesions

**Figure 4:**
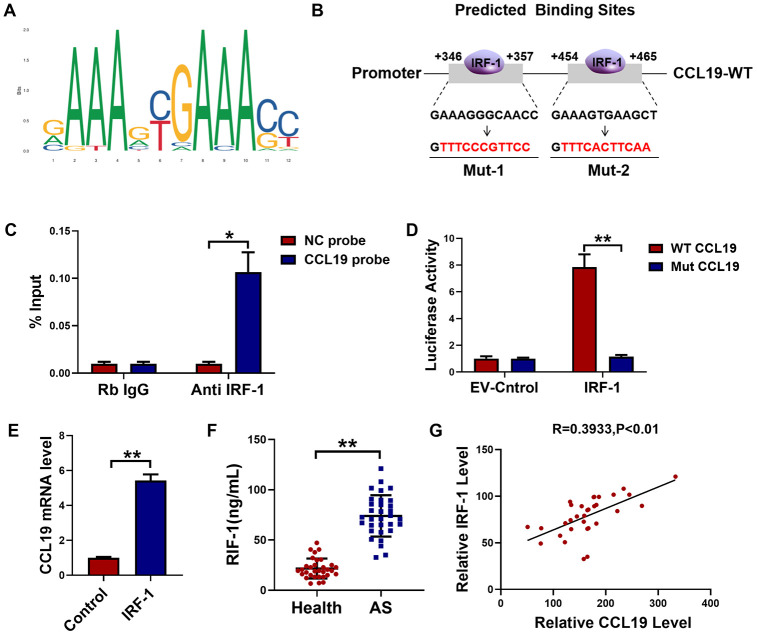
**IRF-1 transcriptionally activated CCL19 expression in VSMCS.** (**A**) Predictive motif of IRF-1 binding to gene promoter. (**B**) The schematic diagram of sequence complementarity between the IRF-1 and CCL19 predicted on JASPAR datasets. (**C**) Chromatin immunoprecipitation (ChIP) assay revealed an association between IRF-1 and CCL19 in VSMCS; IgG served as a negative control; n = 3. (**D**) cells were transfected mut-CCL19/CCL19 with the presence or absence of IRF-1, luciferase activity was measured by dual-luciferase reporter assay system. n=5. (**E**) qRT-PCR detected CCL19 mRNA level after overexpression of IRF-1. n=5. (**F**) Detection of IRF-1 expression in peripheral blood of patients by ELISA.n=32. (**G**) Pearson correlation analysis showed a positive correlation between IRF-1 and CCL19 in peripheral blood. R=0.3933, P<0.01. *P<0.05; **P<0.01.

Through JASPAR datasets (http://jaspar.genereg.net) analysis, we found that IRF-1 may be a transcriptional factor that regulated CCL19, and predicted the potential motif in the promoter region of IRF-1 binding gene (Figure 4A). Database analysis showed that IRF-1 had two possible binding sites in the CCL19 promoter region (Figure 4B). To determine the regulatory relationship between IRF-1 and CCL19, the combination of IRF-1 and CCL19 promoter region was detected by ChIP. Compared with non-specific IgG, the binding of IRF-1 antibody to CCL19 promoter region was significantly increased (Figure 4C). The results show that IRF-1 can directly regulate the expression of CCL19 by binding to the promoter region of CCL19. Next we cloned wild-type or mutated CCL19 promoter into luciferase reporter plasmids. The reporter gene and plasmids of EV and IRF-1 were co-transfected into VSMCS. Luciferase assay showed that IRF-1 binds to the CCL19 (Figure 4D). The results of RT-PCR showed that the overexpression of IRF-1 promoted the mRNA level of CCL19 (Figure 4E). These results demonstrated that IRF-1 activates CCL19 by binding to the region of CCL19 promoter. The expression of IRF-1 in peripheral plasma of patients was detected by ELISA, and the level of IRF-1 was increased (Figure 4F). Pearson correlation analysis showed a positive correlation between IRF-1 and CCL19 in the blood of AS patients (Figure 4G), further explaining the regulatory relationship between them.

### Overexpression of IRF-1 promotes VSMCS pathological phenotype by activating CCL19

**Figure 5:**
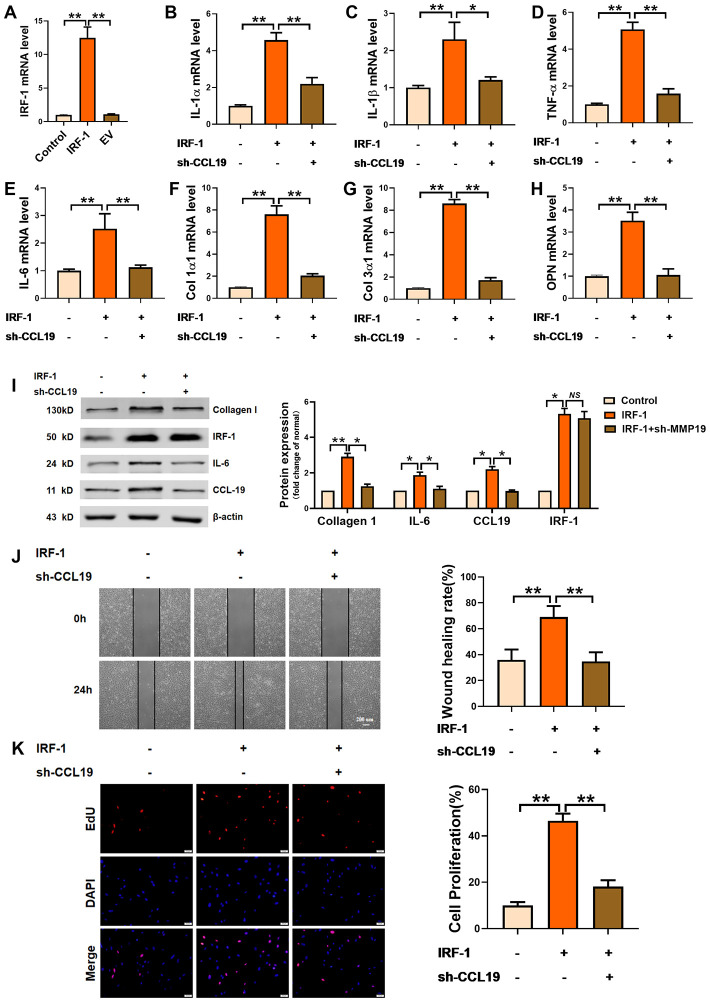
**Effects of IRF-1 on VSMCS though CCL19.** (**A**) Detection of overexpression efficiency of IRF-1 plasmid by qRT-PCR. n=5. Knockdown CCL19 reversed IRF-1 induced mRNA expression of inflammatory cytokine IL1-α (**B**), IL-1β (**C**), TNF-α (**D**), IL-6 (**E**) and the deposition of ECM collagen1 (**F**), collagen3 (**G**) and OPN. (**H**, **I**) Western blot showed the collagen1, IL-6 and CCL19 protein expression induced by IRF-1, but reduced by sh-CCL19. n = 4. Migration (**J**) and proliferation (**K**) of VSMCS of IRF-1 overexpression, with or without sh-CCL19. n = 6. *P<0.05; **P<0.01.

In order to explore the effect of IRF-1 on VSMCS and its regulation on CCL19, an IRF-1 overexpression plasmid was constructed (Figure 5A). Overexpression of IRF-1 increased the expression of inflammation-related factors IL-1 α, IL-1 β, TNF- α and IL-6, while the expression of inflammatory cytokines was inhibited by transfection of sh-CCL19 (Figure 5B–5E). Silencing CCL19 also reduced the expression of extracellular matrix induced by IRF-1 (Figure 5F–5H). As shown by the protein results, overexpression of IRF-1 can significantly increase the protein expression of Collagen1 and IL-6 (Figure 5I). Wound healing and EdU showed that silencing CCL19 could reverse the migration and proliferation of VSMCS induced by overexpression of IRF-1 (Figure 5J, 5K).

### Loss of IRF-1 attenuates AS through modulating CCL19 in *vivo* and in *vitro*

**Figure 6:**
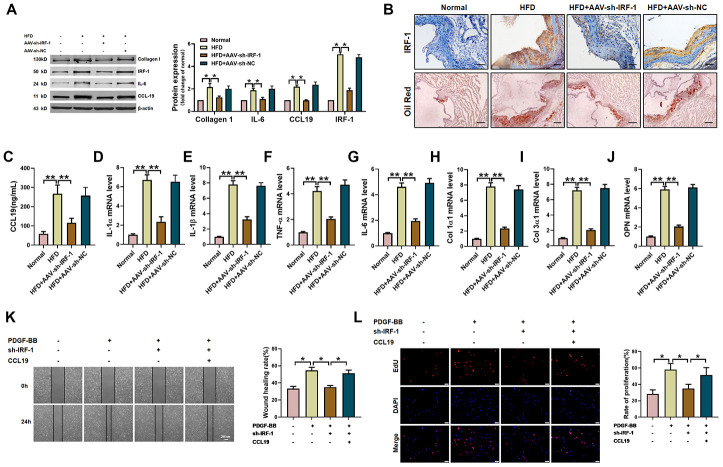
**IRF-1 knockdown inhibits AS of mice and proliferation and migration of VSMCS.** (**A**) Western blot was used to analyze the protein level of IL-6, Collagen 1, CCL19 and IRF-1 in AS mice and AAV-sh-IRF-1 treated mice. β-actin was used as an internal control. n=4 (**B**) Representative images of IHC staining and oil red staining in AS model mice aorta tissues and normal aorta tissues. Bar=50 μm. Knockdown IRF-1 reversed HFD induced mRNA expression of CCL19 (**C**), inflammatory cytokine IL1-α (**D**), IL-1β (**E**), TNF-α (**F**), IL-6 (**G**) and the deposition of ECM collagen1 (**H**), collagen3 (**I**) and OPN (**J**). n=56. In vitro, sh-IRF-1 led migration (**K**) and proliferation (**L**) of VSMCS. n=6. *P<0.05; **P<0.01.

Next, in order to verify the therapeutic effect of silent IRF-1 on atherosclerotic mice, we injected AAV-sh-IRF-1 into the model mice through tail vein. After four weeks of intervention, the aorta of mice was taken for detection. The protein results showed that silencing IRF-1 reduced the expression of IL-6, Collagen1 and CCL19 in high-fat-fed mice (Figure 6A). The results of immunohistochemistry showed that IRF-1 was highly expressed in the arteries of AS model mice, while AAV-sh-IRF-1 significantly silenced the expression of IRF-1. Oil red staining demonstrated a significant increase in lipid in vascular plaques in high-fat-fed mice, and AAV-sh-IRF-1 could alleviate the symptoms of atherosclerosis. (Figure 6B). We found that silencing IRF-1 decreased the expression of CCL19 mRNA (Figure 6C) and inhibited inflammatory cytokines IL-1 α, IL-1 β, TNF- α, IL-6 (Figure 6D–6G) and the ECM deposition (Figure 6H–6J). At the cellular level, silencing IRF-1 decreased the migration and proliferation of VSMCS induced by PDGF-BB, while overexpression of CCL19 restored the ability of cell migration and proliferation (Figure 6K–6L).

## DISCUSSION

Atherosclerotic plaque rupture and thrombosis are the pathological basis of malignant cardiovascular events [13]. Among the various influencing factors, platelet-derived growth factor (PDGF) plays an important role in various mechanisms of cardiovascular disease [14].

Therefore, PDGF-BB was used to simulate the abnormal proliferation and inflammatory secretion of VSMCs under pathological conditions to study the effect and mechanism of IRF-1/CCL19.

The early stage of atherosclerosis is mainly characterized by acute exudative inflammation, the accumulation of low-density lipoprotein droplets, mononuclear macrophage infiltration and creation of foam cells [15]. In the progressive stage, it is mainly characterized by chronic proliferative inflammation, such as massive proliferation of vascular smooth muscle cells and excessive synthesis of extracellular matrix [16, 17]. Proliferation and migration of VSMC is a key step in the formation of new intima after angioplasty. It is well-known that inflammatory and cardiovascular cell-derived MMP2/9 and have critical roles in VSMC migration and proliferation in development of atherosclerosis [18]. Meng et al. [19] demonstrated that deletion of CatK alleviated neointimal hyperplasia by inhibiting inflammation, oxidative stress and the proliferation of VSMC, indicating that CatK is a new therapeutic target for restenosis after intravascular intervention.

**Figure 7:**
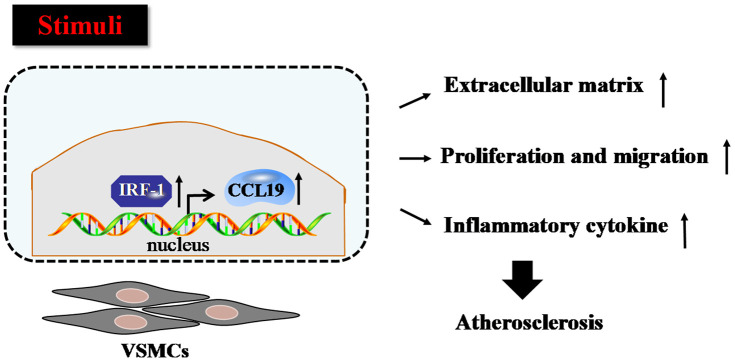
**Proposed model for the mechanism that IRF-1 transcriptionally activates CCL19.** IRF-1 contributed to extracellular matrix deposition, proliferation and migration, inflammatory cytokines secretion of VSMCs during atherogenesis by increasing CCL19 transcription.

Firstly we verified that CCL19 highly expressed in patients with atherosclerosis. As a soluble chemokine released during platelet activation and a sensitive marker of blood hypercoagulable state, CCL19 also induce monocytes to gather to the lesion site and play an important role in promoting inflammatory response [20]. It has been reported that the expression of CCL19 is increased in human atherosclerotic carotid artery disease [21], and it has been found that the expression of CCL19/CCL21/CCR7 in atherosclerotic coronary artery tissue increases, and promotes atherosclerosis by regulating the adhesion and migration of human monocytes [22]. In this study, the results showed that the level of serum CCL19 in AS group was higher than that in control group, and the level of CCL19 in arterial tissue of AS model mice also increased significantly, indicating that CCL19 may be a blood biomarker for diagnosis of AS. In *vitro* experiments, we found that overexpression of CCL19 promoted inflammatory secretion, ECM deposition, proliferation and migration of VSMCS. Through the analysis of bioinformatics website, we found that IRF-1 may be the upstream regulatory factor of CCL19. We predicted the possible binding sequence of transcription factor IRF-1 to CCL19 promoter and verified the transcriptional regulation of IRF-1 on CCL19 by ChIP and luciferase reports. These findings attempt to elucidate the regulatory mechanisms of CCL19 and IRF-1 in atherosclerosis (Figure 7).

As a key immune stress molecule, it has been found that IRF-1 plays an important role in regulating hematopoietic development [23]. Testa et al. [24]found that the number of immature granulocyte progenitor cells in the bone marrow of IRF-1 knockout mice increased significantly. In contrast, the number of mature granulocytes decreased significantly, indicating that IRF-1 also plays an important role in the regulation of granulocyte differentiation and maturation. Meanwhile, IRF-1 is an important part of interferon (Interferon, IFN) pathway. After binding to the membrane receptor, IFN γ phosphorylates JAK1 and STAT1, in turn, and then STAT1 enters the nucleus to activate the expression of IRF-1 [25]. Our results showed that silencing IRF-1 can significantly inhibit the inflammatory response and extracellular matrix deposition in *vitro* and in *vivo*, and inhibited the migration and proliferation of VSMCS. In general, our research demonstrated that IRF-1 and CCL19 are involved in the course of atherosclerosis, which may be used as diagnostic markers and new therapeutic targets.

## MATERIAL AND METHODS

### Clinical blood samples

From August 2017 to August 2018, 32 AS patients with atherosclerosis were diagnosed by angiography in the second affiliated Hospital of Harbin Medical University as the case group, and the healthy control group was 32 patients of the same age from the physical examination center of the hospital at the same time. There were 15 males and 17 females with an average age of 64 years. The levels of CCL19 and IRF-1 in peripheral blood of the two groups were detected by ELISA. This experimental study was approved by the Ethics Committee of Harbin Medical University, and all participants signed informed consent forms.

### Animals

A total of 30 eight-week-old male SPF ApoE-/- mice (20-22 g) were purchased from Cavens Experimental Animal Co., Ltd., and were raised in the SPF animal room of the Clinical Research Center of the second affiliated Hospital of Harbin Medical University. The mice were divided into three groups: normal feeding group, high-fat diet group and tail vein injection of adeno-associated virus 9-sh-IRF-1 (AAV9-sh-IRF-1) treatment group. The high-fat diet and standard diet was bought form Medicience Ltd.(Jiangsu, China). AAV9-sh-IRF-1 and AAV9-sh-NC were manufactured by Jikai Co. (Shanghai, China). Atherosclerosis was induced by feeding high-fat diet with light-dark cycle for 12 hours for 8 weeks. The animal experiment was approved by the Medical Ethics Committee of Harbin Medical University (Table 1).

**Table 1:** Rat chow composition (g/kg).

**Ingredients**	**Standard diet**	**High fat diet**
Milk	/	145
Sucrose	/	290
Wheat flour	216	188
Corn flour	324	237
Margarine	/	143.3
Palm oil	50	55
Cellulose	/	30
Mineral salts	20	20
Vitamins	5	5
Water	385	/

### Cell culture

Human aortic vascular smooth muscle cells (HA-VSMCs) were purchased from the Chinese Academy Cell Bank (Shanghai, China). Under the condition of 5% CO_2_ at 37° C, with DMEM high glucose medium (10% FBS) the cells were cultured in the incubator. The cells stimulated by PDGF-BB (10 ng/ml) for 48 h, which was purchased from R&D Systems (Abingdon, UK). The logarithmic growth phase cells were used in the experiment.

### ELISA assay

The levels of CCL19 and IRF-1 in blood were detected by enzyme-linked immunosorbent assay (ELISA). All the kits were bought from Shanghai Yaji Biotechnology Co., Ltd. Fasting venous blood was drawn from all subjects, and all operations were completed according to the instructions of the kit in the standard laboratory.

### RNA transfection

The HA-VSMCS were plated in dishes until the cell density reached 80% confluency to transfect. The plasmids were transfected by Lipofectamine 2000 (Invitrogen, Carlsbad, CA). Short hairpin RNA (shRNA) was used to silence CCL19 or IRF-1. The plasmid of CCL19/sh-CCL19 and IRF-1/sh-IRF-1 were constructed from Genechem (Shanghai, China).

CCL19 sh-RNA Sequence: CCGGGCCCTGGGTAGAACGCATCATCTCGAGATGATGCGTTCTACCCAGGGCTTTTTG. IRF1 sh-RNA Sequence: CCGGTTCACACAGGCCGATACAAAGCTCGAGCTTTGTATCGGCCTGTGTGAATTTTTG.

### Immunohistochemical staining

Paraffin sections of arteries were dewaxing to water in xylene and descending series of ethanol. We penetrated sections using 0.5% Triton X-100. After 3 times wash, we blocked sections with 50% goat serum. For immunohistochemical staining, sections were incubated with CCL19/IRF-1 antibody overnight. We incubated the sections using secondary antibody followed by DAPI staining. The sections were photographed by light scope under an IX73 fluorescence microscope (Olympus, Valley, PA).

### EdU assay

Using EdU Cell Proliferation Kit (RiboBio, Guangzhou, China) to test proliferation. HA-VSMCS were seeded in 24-well plates for transfection. Cells were added with 200uL 50 uM EdU and incubated for 2 h. Apollo Dye Solution(red) were used to stain proliferating cells, nucleic acids were stained with DAPI (blue)according to the protocols and then photographed.

### Wound-healing assay

To test the cell migration ability of HA-VSMCS, cells were seeded in 6-well plates until the cells formed a confluent monolayer, then scratched using a 100 μl pipette tip. The scratch was photographed using phase-contrast microscopy an at 0, 24h. The relative wound size was analyzed by imageJ.

### Luciferase reporter assays

HA-VSMCS (1 × 10^5^) were plated in a 24-well plate before transfection. Next day, cells were co-transfected with the empty vector (EV) or the IRF-1 plasmid and wild type (WT) or mutant (Mut) CCL19 promoter plasmid-based firefly luciferase reporter. Luciferase activities were measured using the dual luciferase reporter assay kit (Promega, USA) after 48 h.

### Western blot

Cells and tissues protein were lysed with RIPA buffer (Beyotime, Jiangsu, China). All protein was run on a 10% SDS-polyacrylamide gel. Primary antibody against CCL19, Collagen 1, IL-6, IRF-1 and β-actin were purchased from Affinity Bioscience (Jiangsu, China), with β-actin as an internal control. Odyssey Infrared Imaging System (Odyssey CLx, Biosciences, USA) was used to detect immunoreactivity.

**Chromatin Immunoprecipitation (ChIP)**

ChIP experiment uses ChIP-IT Express Enzymatic kit, purchased from Active Motif company in the United States, the experimental steps are carried out in strict accordance with the product instructions: DNA purification and qPCR analysis. The CCL19 promoter region, which contains two IRF-1 binding sites was detected. The experimental samples are analyzed by Input samples, and the data are quantized as the percentage of Input.

### Statistical analysis

All the data were analyzed and compared with Graphpad 7.0 software, and all the experimental data were expressed as mean ±SD. Student *t* test was used for statistical analysis between the two groups, one-way ANOVA analysis was used to compare the data between the two groups, and Pearson correlation test was used to analyze the correlation. P<0.05 was considered significance.
